# Enhancing Specific Disruption of Intracellular Protein Complexes by Hydrocarbon Stapled Peptides Using Lipid Based Delivery

**DOI:** 10.1038/s41598-017-01712-5

**Published:** 2017-05-11

**Authors:** D. Thean, J. S. Ebo, T. Luxton, Xue’Er Cheryl Lee, T. Y. Yuen, F. J. Ferrer, C. W. Johannes, D. P. Lane, C. J. Brown

**Affiliations:** 1 p53 Laboratory, A*STAR, Singapore, Singapore; 2 Organic Chemistry, ICES, A*STAR, Singapore, Singapore

## Abstract

Linear peptides can mimic and disrupt protein-protein interactions involved in critical cell signaling pathways. Such peptides however are usually protease sensitive and unable to engage with intracellular targets due to lack of membrane permeability. Peptide stapling has been proposed to circumvent these limitations but recent data has suggested that this method does not universally solve the problem of cell entry and can lead to molecules with off target cell lytic properties. To address these issues a library of stapled peptides was synthesized and screened to identify compounds that bound Mdm2 and activated cellular p53. A lead peptide was identified that activated intracellular p53 with negligible nonspecific cytotoxicity, however it still bound serum avidly and only showed a marginal improvement in cellular potency. These hurdles were overcome by successfully identifying a pyridinium-based cationic lipid formulation, which significantly improved the activity of the stapled peptide in a p53 reporter cell line, principally through increased vesicular escape. These studies underscore that stapled peptides, which are cell permeable and target specific, can be identified with rigorous experimental design and that these properties can be improved through use with lipid based formulations. This work should facilitate the clinical translation of stapled peptides.

## Introduction

Interest in chemical modifications that enable the translocation of peptides into cells is spurred on by the increasing use of biologics, peptides, proteins and their mimetics as tools in research and therapeutic development^1–3^. Several different covalent linkages have been used to stabilize the receptor bound helical conformations of peptides in solution^4^. The hydrocarbon ‘staple’, which consists of the coupling together of 2 olefin bearing non-natural amino acids located at suitable positions along the peptide, is one such increasingly used modification and has been shown to enable cellular uptake, increased proteolytic stability and improved distribution in animal models of a variety of peptides^5–7^. One of the main features of stapled peptides is their ability to potently bind the large and relative flat polar surface areas that are found in a large majority of protein-protein interactions (PPIs)^8^, which are intractable to small molecules. A large proportion of therapeutically relevant PPIs are intracellular and many are mediated by an α-helix in one of the proteins constituting the interface. This makes them attractive targets for the development of stapled peptides^9^.

The p53: Mdm2 interaction is a clinically relevant example of a PPI mediated by an α-helix^10^. p53 is a tumor suppressor protein that is frequently inactivated in human cancers by over-expression of Mdm2, which targets p53 for proteosomal degradation via its E3 ubiquitin ligase activity^11^. Inactivation of p53 allows malignant cells to escape cell cycle arrest and apoptosis^11^. The unfolded transactivation domain of p53 forms a helix when it binds Mdm2, where upon 3 conserved residues (F19, W23, L26) are buried into a hydrophobic cleft upon the surface of Mdm2^12^. Several stapled peptides have been designed by several groups against Mdm2 using binding sequences either derived from the known p53 binding epitope or isolated using phage display experiments^13, 14^. A stapled peptide antagonist (ALRN-6924) of the Mdm2:p53 interaction is undergoing phase 1 clinical trials (ClinicalTrial.gov). Extensive research elsewhere has also been directed towards developing stapled peptides against a variety of targets such as EZH and Bcl-2^15–17^.

The potential of stapled peptides to be developed as therapeutic compounds have been restricted by their lack of ubiquitous cellular permeability^18^, avid serum binding^7^, and indications that their biological and *in vivo* activities are the results of non-specific modes of actions, which are target independent^19^. This brings into question whether or not stapled peptides engage their intracellular targets and enter cells in the absence of cell membrane disruption, which gives rise to cytotoxicity and other nonspecific signals that can be falsely concluded to be mechanistically on target^19^. To address these concerns we synthesized a library of stapled peptides, using the helically **s**tabilized **M**dm2 binding pept**ide** (sMTIDE-02) developed by ourselves as a template^6^, and stringently screened for compounds with improved cellular activity and negligible cytotoxicity, as a function of cell membrane disruption. The cellular permeability and mechanistic action of these peptides was then confirmed by measuring the disruption of intracellular p53:Mdm2 complexes using a live cell protein-protein interaction BRET (bioluminescent resonance excitation transfer assay) based assay^20^. The top ranked compound identified however only showed a marginal improvement in its cellular potency compared to sMTIDE-02, whilst still exhibiting substantial serum binding. To overcome these hurdles and enhance its cellular delivery, various lipid based preparations were then explored in combination with the newly identified optimized stapled peptide^21–26^. This lead to the identification of a pyridinium amphiphile cationic lipid formulation that enhanced the activity of the p53 activating stapled peptide by an order of magnitude, principally through more efficient vesicular escape into the cytoplasm.

## Results and Discussion

### High Stringency Library Screening for Stapled p53 Re-Activating Peptides with Improved Cellular Activity and Decreased Cyto-toxicity

**Figure 1:**
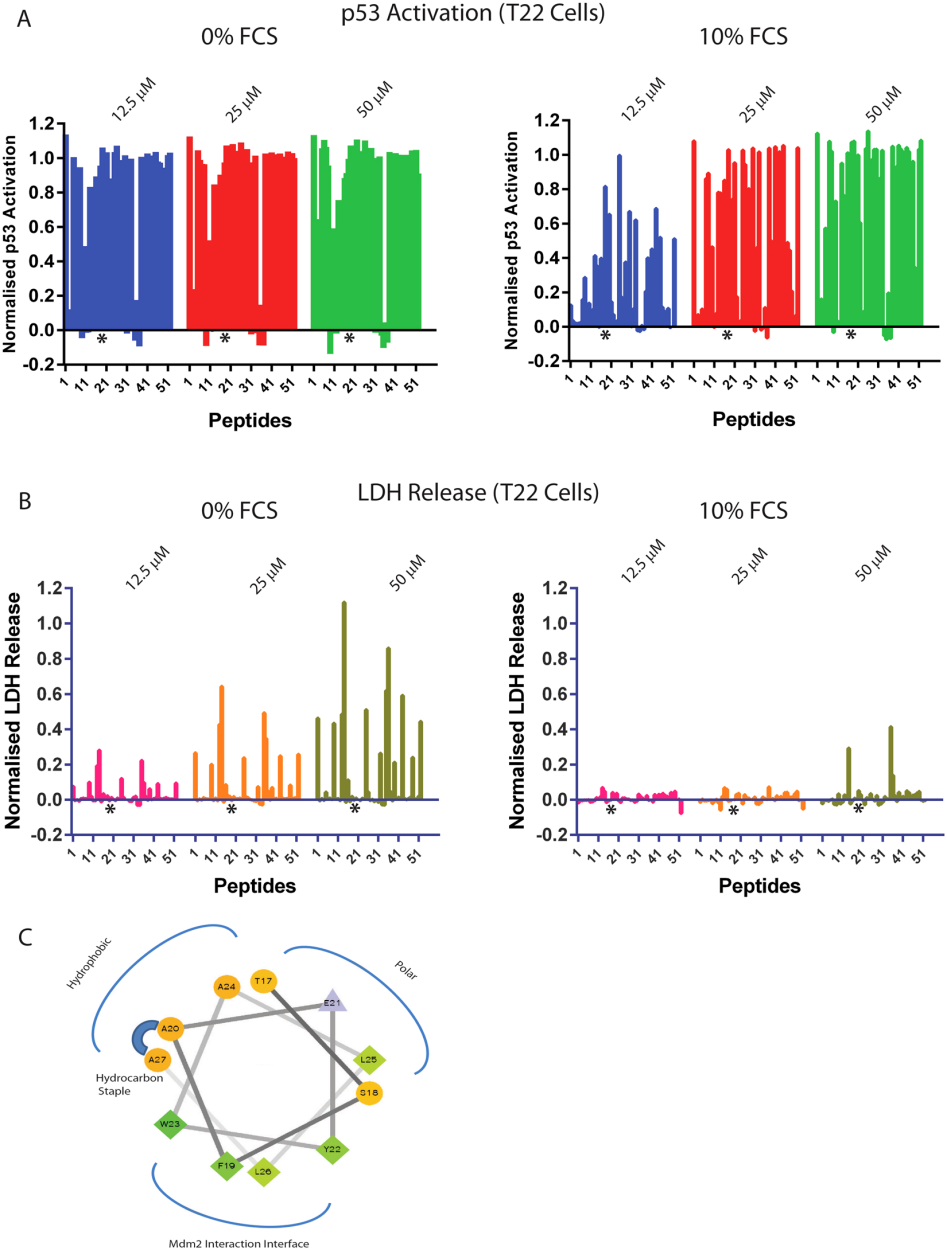
(**A**) Results for the screening of a 52 membered stapled peptide library using the T22 p53 reporter cell based assay. The library was screened either in the presence (10%) or absence of FCS (fetal calf serum) at 3 different concentrations to capture weak and strong inducers of p53 activation. (**B**) Results for screening the library using the CYTO-TOX LDH release assay (Promega) to assess for membrane destabilization. The library was screened either in the presence (10%) or absence of FCS at three different concentrations to capture weak and strong LDH releasers. (**C**) A helical wheel representation of the sMTIDE-02 template sequence used for the design of the peptide library. The peptides in the library are amphipathic in nature with a hydrophobic aliphatic chain running down one face of the helix, the hydrophobic Mdm2 conserved binding motif running down the other and the remainder of the molecule being slightly more polar in character. * indicates the location of VIP-82 amongst the stapled peptides screened.

A library of stapled peptides targeting the p53:Mdm2 interaction, using sMTIDE-02 as a template, was synthesized and assessed for binding to Mdm2 using a competitive fluorescence anisotropy (FA) assay, for p53 activity in a reporter cell line (T22 cells) and for their ability to disrupt cell membranes of the p53 reporter cells using a LDH (lactate dehydrogenase) release assay (Fig. 1A and B). LDH release was measured within 2 hours after compound treatment to differentiate cell membrane disruption from the later stages of apoptosis, and from effects related to the onset of p53 transcriptional activity. The p53 reporter assay and LDH release assays were also performed in the presence and absence of serum, the former to reflect more physiologically relevant conditions and the latter to enable identification of sequences unaffected by sequestration to components in the serum. In the absence of serum a significant proportion of the peptide library induced p53 activity (~ over 50% of the screened peptides caused induction of p53 activity over a normalized value of 0.5) at the lowest treatment concentration used (12.5 µM), whilst in the presence of 10% serum a dramatic overall decrease in the potency of the library was observed indicating that serum binding is a common property of stapled peptides (Fig. 1A). In addition, a large proportion of the peptides (~20%) in the library also exhibited cytotoxicity (LDH leakage) in the absence of serum (Fig. 1B), which was significantly reduced in the presence of serum (to ~ 5%). This confirms that LDH leakage, which is a proxy measurement for cell membrane disruption, correlates to the increased concentration of free peptide when it is unbound to serum. sMTIDE-02 and ATSP-7041, reported by ourselves^6^ and Aileron^7^ independently of each other, were also included in the library and confirmed to disrupt the Mdm2:p53 complex *in vitro* and to activate p53 in the T22 cell based reporter assay. However, under no serum conditions, in agreement with published findings^19^, both peptides also induced LDH leakage indicating destabilization of the cell membrane alongside p53 activation, an undesirable activity indicating the occurrence of p53 independent effects or potential off-target mechanistic activation by cellular stress.

**Figure 2:**
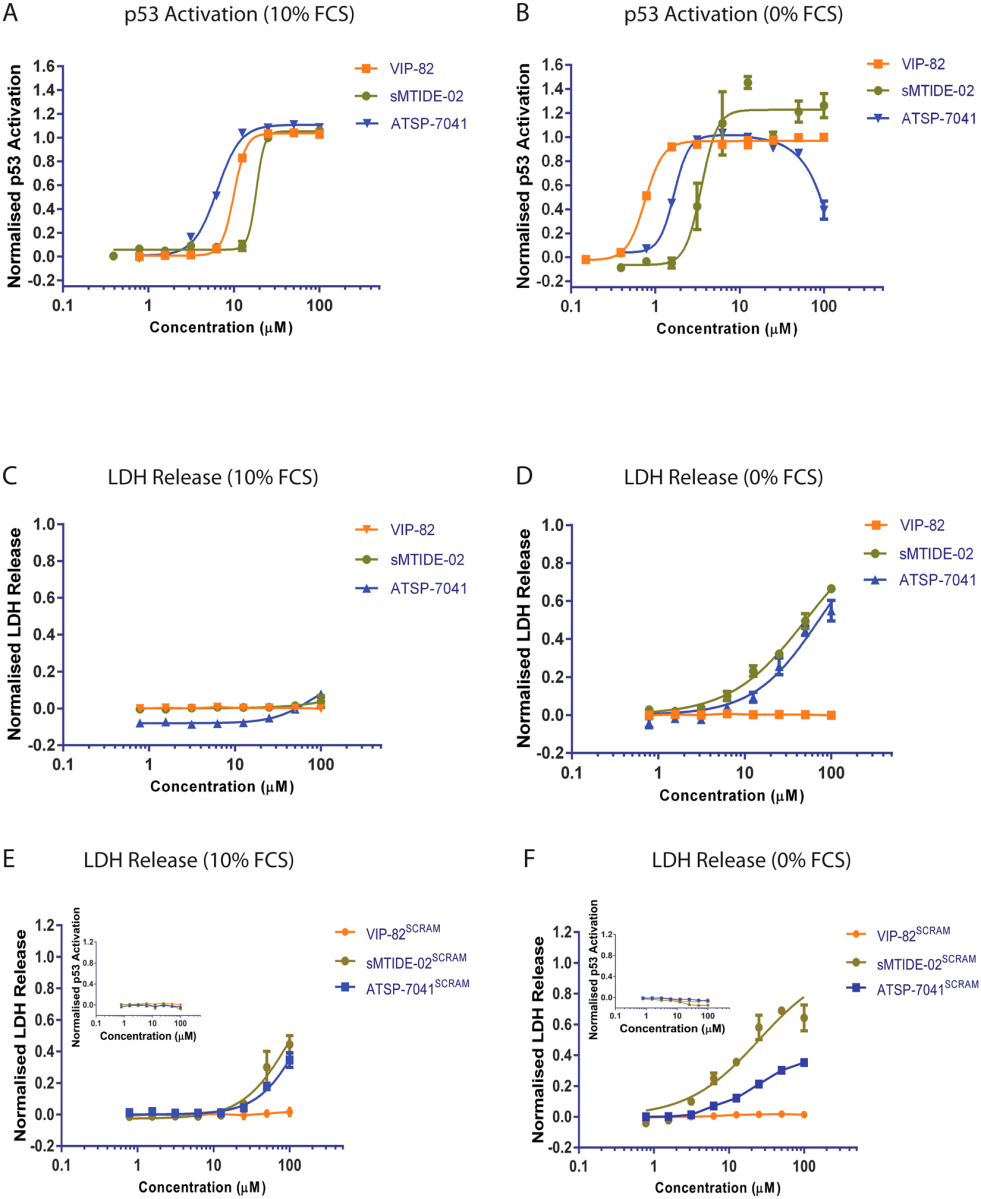
sMTIDE-02, ATSP-7041 and VIP-82 were titrated on to T22 p53 reporter cells and p53 transcriptional activation assessed (**A**) in the presence (10% FCS) or (**B**) absence of serum. (**C** and **D**) Identical titrations were performed on T22 cells and LDH release measured. Scrambled variants of sMTIDE-02, ATSP-7041 and VIP-82 (designated with scram in superscript) were synthesized, where the residues involved in the conserved binding motif (F19, W23, L26) were interchanged with each other to abrogate binding to p53. sMTIDE-02^SCRAM^, ATSP-7041^SCRAM^ and VIP-82^SCRAM^ were titrated on to p53 reporter T22 cells (**E**) in the presence (10%) and (**F**) absence of serum and assessed for p53 activation and LDH release. p53 activations profiles are shown in the insets.

To choose the peptide with the most desirable properties i.e. high induction of p53 under physiological conditions and negligible cyto-toxicity, we carefully selected the compound that induced the highest amount of p53 activity in the presence of serum and also caused no measurable LDH release in the absence of FCS. This resulted in the identification of the VIP-82 compound (Ac-KK-Ahx-TSFR_8_EYWALLS_5_ENF-NH_2_, R_8_ = (R)-2-(7′octenyl) alanine and S_5_ = (*S*)-2-(4′-pentenyl) alanine), which differed from the initial sMTIDE-02 template sequence with a N-terminal di-lysine extension via an aminohexinoic linker to improve solubility and a C-terminal extension (ENF) to exploit a newly discovered interaction site on the surface of Mdm2^27^. In comparison to the previously reported stapled Mdm2 binding peptides (sMTIDE-02 and ATSP-7041), the K_d_ determined for VIP-82 (24.4 ± 2.3 µM, Table S1) binding to Mdm2 was marginally stronger than that for sMTIDE-02 (34.35 ± 2.0 µM, Table S1), but was significantly weaker than the K_d_ determined for ATSP-7041 (7.4 ± 1.5 µM, Table S1). However VIP-82 (EC_50_ = 0.8 ± 0.1 µM) was more efficacious in inducing p53 activity in the T22 p53 reporter cells than both sMTIDE-02 and ATSP-7041 (EC_50_s of 3.5 ± 0.3 µM and 1.7 ± 0.1 µM, respectively) in the absence of serum (Fig. 2A and B, Table 1). EC50s were also determined in the presence of 10% FCS, which revealed that the ATSP-7041 was the least effected by serum binding amongst the 3 peptides (Table 1) and the most potent activator of p53 under serum conditions (Fig. 2A and B). However VIP-82 more importantly, unlike ATSP-7041 and sMTIDE-02, did not cause any measurable LDH release in the absence of serum indicating decreased cellular toxicity arising from cell membrane disruption, as well as eliminating this as a potential mode of p53 activation for VIP-82 (Fig. 2C and D).

**Table 1:** p53ACT_50_ and LDH_50_ values were determined from 4 parameter fits to 10 point titration curves.

Peptide	P53 Activation (T22 Cells, µM)		LDH Release (T22 Cells, µM)	
	0% FCS	10% FCS	0% FCS	10% FCS
sMTIDE-02	3.5 ± 0.3	18.2 ± 0.7	50.5 ± 1.7	No release
ATSP-7041	1.7 ± 0.1	6.4 ± 0.2	71.8 ± 4.8	No release
VIP-82	0.8 ± 0.1	10.0 ± 0.1	No release	No release
sMTIDE-02^SCRAM^	No Activity	No Activity	25.1 ± 2.8	105 ± 8.0
ATSP-7041^SCRAM^	No Activity	No Activity	191.1 ± 20.1	149.0 ± 10.1
VIP-82^SCRAM^	No Activity	No Activity	No Release	No Release

Interestingly, the onset of p53 activity for ATSP-7041 and sMTIDE-02 under serum free conditions still occurred at concentrations below the threshold required for the induction of LDH release, indicating that gross destabilization of the membrane by these compounds also does not directly elicit p53 activity. To conclusively confirm that the induction of p53 transcriptional activity in T22 p53 reporter assays is not driven by cellular stress mechanisms e.g. cell membrane perturbation or other unknown factors, negative control peptides were carefully designed for sMTIDE-02, ATSP-7041 and VIP-82. To achieve this, the 3 residues responsible for the interaction of the peptides with Mdm2 were scrambled, in order to abrogate binding to Mdm2, and the remainder of the sequence left unchanged. Scrambling was used rather than the introduction of alanine point mutants to generate control peptides, as this ensured minimal perturbation of the physiochemical parameters and maintained the amphipathicity of the stapled peptides (Fig. 1C), a variable critical in the cellular entry of stapled peptides^28^. The new control analogues (sMTIDE-02^SCRAM^, ATSP-7041^SCRAM^ and VIP-82^SCRAM^) showed no binding against Mdm2 in the FA competition assay and induced no p53 activity when tested on T22 cells (Table 1 and Table S1). However, both sMTIDE-02^SCRAM^ and ATSP-7041^SCRAM^, unlike their unscrambled counterparts, also induced LDH release in the presence of serum as well as in its absence. (Fig. 2E and F, Table 1). The ability of sMTIDE-02^SCRAM^ and ATSP-7041^SCRAM^ to efficiently disrupt cellular membranes, without inducing cellular p53 activity or binding to Mdm2 *in vitro*, confirms that p53 activating peptides act mechanistically through the disruption of the intracellular Mdm2:p53 complex and not through membrane disruption.

### Stapled Peptides Specifically Disrupt the Intracellular p53:Mdm2 Complex within Live Cells

To verify that the molecular mechanism of p53 activation was due to intracellular engagement of Mdm2 by the stapled peptides (VIP-82, ATSP-7041 and sMTIDE-02) and through their disruption of the Mdm2:p53 complex a nanoBRET (Bioluminescence Resonance Excitation Transfer, PROMEGA) live cell assay was used^20^. The p53:Mdm2 NanoBRET System is a proximity-based assay that can detect protein interactions by measuring energy transfer from a bioluminescent luciferase protein donor (nanoLUC, Promega), which is fused to p53, to a fluorescent protein acceptor (HaloTag, PROMEGA) that is joined to Mdm2. Both constructs are transiently transfected into a cell population, which are then treated with a cell permeable fluorogenic substrate that covalently labels the HaloTag, and causes a BRET signal to be generated. This signal is only generated when the Mdm2 and p53 interact with each other and bring the nanoLUC and HaloTag into the local vicinity of each other, enabling the transfer of the bioluminescence signal to the fluoorphore. When the Mdm2 and p53 complex is disrupted by a cell accessible molecule the fusion proteins are no longer in proximity to each other and the signal is lost.

**Figure 3:**
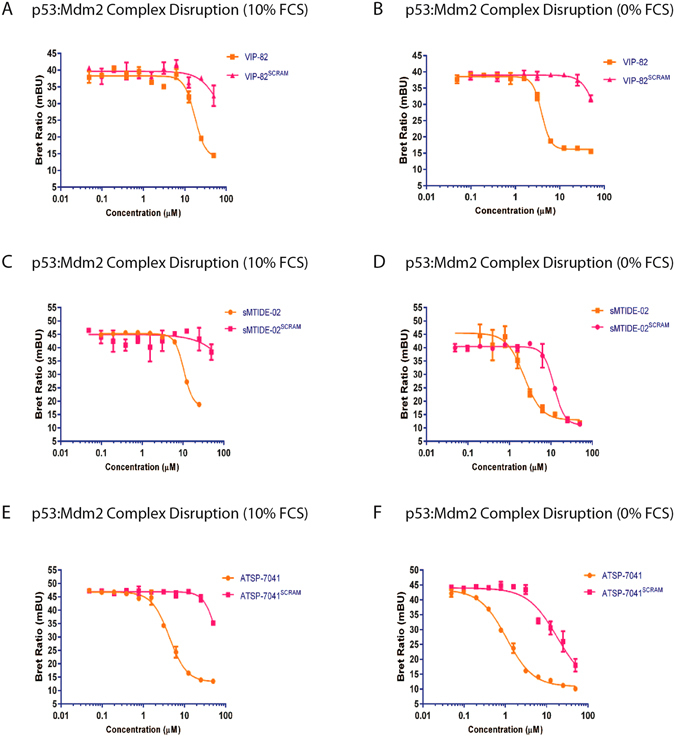
Disruption of intracellular Mdm2:p53 complexes were assessed using the nanoBRET assay (Promega) and IC50s were determined. Titrations were carried out in pairs between the active stapled peptide and the scrambled control variant to determine the window of specificity between targeted disruption of the Mdm2:p53 complex and the onset of non-specific cellular effects: VIP-82 and VIP-82^SCRAM^ (**A**) in 10% FCS and (**B**) 0% FCS, sMTIDE-02 and sMTIDE-02^SCRAM^ (**C**) in 10% FCS and (**D**) 0% FCS, and ATSP-7041 and ATSP-7041^SCRAM^ in (**E**) 10% FCS and (**F**) 0% FCS. Titrations were performed on HEK293 cells and nanoBRET signal was measured after 4 hours.

The p53 activating stapled peptides (sMTIDE-02, ATSP-7041, VIP-82), upon titration of HEK293 cells in physiologically relevant high serum conditions, all efficiently disrupted the transfected intracellular Mdm2:p53 complexes (Fig. 3A,C and E) in the absence of LDH release, (Figure S1) confirming that all three compounds were cell permeable and that mechanism of p53 action is indeed through specific inhibition of Mdm2. The p53:Mdm2 NanoBRET live cell experiments were then repeated in serum free conditions where a substantial improvement in the IC50s of the stapled peptides occurred, with 4.5-fold, 4-fold and 9-fold improvements seen for the IC50s of VIP-82, ATSP-7041 and sMTIDE-02, respectively (Fig. 3B,D and F, Table 2). These findings further confirm the attenuating effect of stapled peptide sequestration by binding to serum components. However, ATSP-7041 and sMTIDE-02, apart from showing improved cellular permeability in the absence of serum components, also induced significant amounts of LDH release, unlike VIP-82 which still remained inert (Figure S1). However, as observed in the T22 cell based p53 reporter titrations, disruption of the intracellular Mdm2:p53 complex occurred at concentrations below that required for LDH release, providing more evidence that cell membrane disruption has no role in the activation of p53.

**Table 2:** NanoBRET IC50 values determined from 4 parameter fits to 10 point titration curves.

Peptide	Mdm2:p53 Complex Disruption (HEK293 Cells, µM)	
	0% FCS	10% FCS
sMTIDE-02	2.3 ± 0.2	17.9 ± 0.2
ATSP-7041	1.1 ± 0.1	4.3 ± 0.2
VIP-82	3.9 ± 0.2	17.5 ± 1.3
sMTIDE-02^SCRAM^	11.9 ± 0.5	NA
ATSP-7041^SCRAM^	17.92 ± 1.4	NA
VIP-82^SCRAM^	NA	NA

The inactive scrambled analogues were also titrated in the nanoBRET assay and caused negligible inhibition of Mdm2 binding to p53 under high serum conditions. However in the absence of serum, both ATSP-7041^SCRAM^ and sMTIDE-02^SCRAM^ did induce disruption of the Mdm2:p53 complex (Fig. 3D and F), which did not occur for VIP-82^SCRAM^. At similar treatment concentrations both compounds also induced significant amounts of LDH release (Figure S1), implying that disruption of the cellular membrane enables the release of Mdm2:p53 complexes into the surrounding cell media, whereby the subsequent dilution causes their dissociation and a concomitant decrease in the BRET signal. However, more importantly, this nonspecific effect elicited by the scrambled analogues occurs at concentrations significantly above the IC_50_s determined for the active peptides.

### Enhancing Cellular Delivery and Intracellular Activity of VIP-82 using a Pyridinium Cationic Based Lipid Formulation

VIP-82, despite a 2-fold improvement in its affinity for Mdm2 over the library template sequence (sMTIDE-02) and the elimination of its ability to lyse cellular membranes, only showed a marginal improvement in its potency to reactivate p53 (Fig. 2A,B and D, Table 1). In addition, VIP-82 also displayed no significant decrease in its serum binding compared to sMTIDE-02, leading to substantial decreases in its biological activity in the presence of serum (Fig. 2A, Table 1). To address these limitations we decided to investigate several different lipid based formulations: NanoCargo (Tecrea, www.tecrea.co.uk), **s**terically **s**tabilized **m**icelles (SSM)^22, 23^ constituted from mPEG-2K-DSPE^7^ (Avanti Polar Lipids, Inc.) and Saint PhD (Synovolux, www.synvolux.nl)^24, 26^. Each of these formulations can either form micelles or liposomes, which either encapsulate or interact with their respective cargo, in this case VIP-82, and prevent the compound being sequestered by serum components and thus potentially enhancing the compounds cellular delivery.

**Figure 4:**
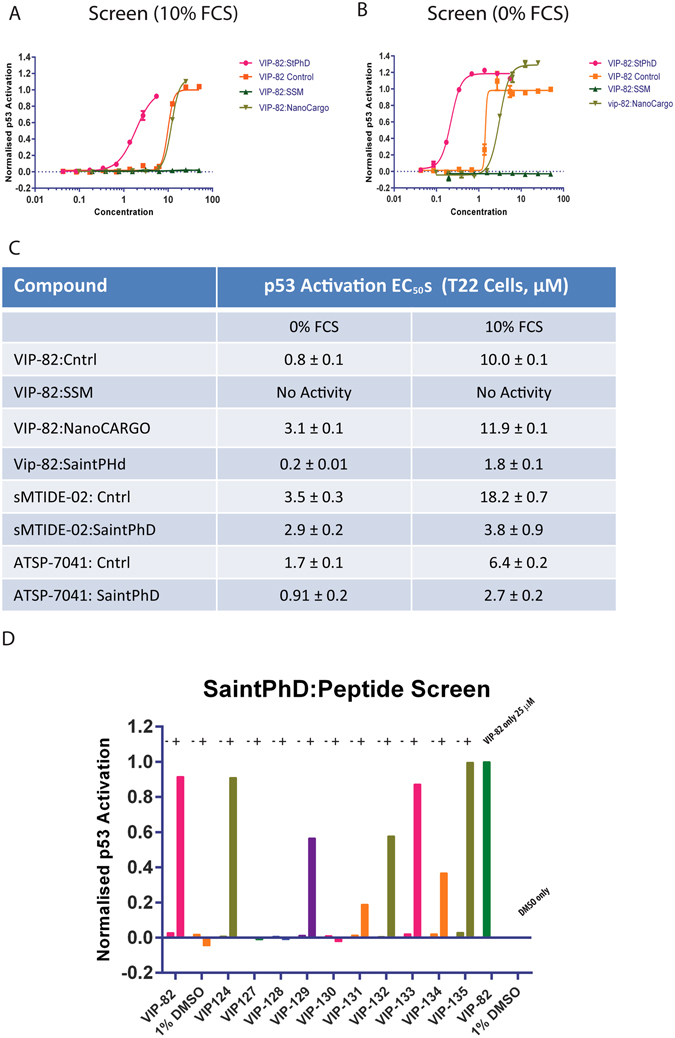
VIP-82 was formulated with either Saint PhD (Synvolux, Netherlands), Nanocargo (Tecrea, UK) or mPEG-2K-DSPE (Avanti Polar Lipids, Inc.) and titrated onto T22 p53 reporter cells in the (**A**) presence or (**B**) absence of serum. (**C**) Table displaying EC_50_s of p53 activation, determined from titration curves produced by the p53 reporter assay, for sMTIDE-02, ATSP-7041 and VIP-82 either alone or formulated with SaintPhD. EC_50_ values were also determined either in the presence or absence of fetal calf serum. In addition results for VIP-82 formulated with Nanocargo and DSPE-mPEG2K derived SSMs are also shown. (**D**) A subset of stapled peptides were selected and either formulated with SaintPhD (+) or without (**−**) and screened at a concentration of 6 µM in the presence of 10% fetal calf serum (FCS) using T22 p53 reporter cells.

VIP-82 was initially titrated onto p53 reporter T22 cells in complex with SaintPhD, a proprietary lipid formulation that consists of a pyridinium cationic lipid and DOPE (dioleoylphosphatidylethanolamine) mixture. EC_50_s of p53 activation were then determined in the absence and presence of serum, which revealed a dramatic 4-fold and 5-fold improvement over the non-formulated VIP-82, respectively (Fig. 4A and B). These results suggest that SaintPhd primarily enhances the uptake of the peptide into cell and that the serum protective effect is smaller. The two other formulations were also assessed, both of which caused no significant improvement in the potency of VIP-82 in the p53 reporter system (Fig. 4A,B and C). In fact, the SSM formulation completely abrogated induction of p53 by the VIP-82 peptide, whilst the NanoCargo formulation had a negligible effect on the peptide’s activity in the presence of FCS and even marginally attenuated it in the absence of serum (Fig. 4B). The SSM formulation consists of micelles constituted from the pegylated lipid mPEG-2K-DSPE, where the PEG part of the molecules form a palisade around a buried hydrophobic lipid core^29^. The abrogation of the p53 activity by the SSM formulation implies that VIP-82 is strongly sequestered and is either interacting with the PEG palisade or the hydrophobic cores of the particles, which in turn prevents any potential binding to Mdm2. On the other hand, NanoCargo only attenuated the activity of VIP-82 in the absence of serum and had no effect on the peptide in the presence of serum, suggesting that it also interacted with the NanoCargo formulation but much less strongly.

To ensure that the p53 transcriptional activity of VIP-82:SaintPhd was specific and on-target LDH release assays were performed that showed little measurable cell membrane disruption (Figure S2). Small amounts of release were however detected at a peptide concentration of 6 µM in the absence of serum. In addition VIP-82^scram^ was formulated and tested under identical experimental conditions to VIP-82:SaintPhD, which revealed that it did not induce any p53 activity or LDH release (Figure S2) confirming specific reactivation of p53. ATSP-7041 and sMTIDE-02 were also re-constituted with SaintPhD, where interestingly sMTIDE-02 showed no improvement in the absence of serum but did show a significant increase in potency in the presence of serum demonstrating the SaintPhD can prevent serum sequestration in a sequence specific manner, whilst ATSP-7041 on the other hand exhibited marginal improvements under both conditions (Fig. 4C). These results emphasize that the dramatic improvements observed for VIP-82 in cellular potency with respect to lipid formulation are also sequence dependent. A subset of peptides were further selected and screened against T22 p53 reporter cells, either formulated with or without SaintPhD, at a concentration where none of the compounds activated p53. Several peptides were potentiated in a manner similar to VIP-82 demonstrating that other sequences can act synergistically with SaintPhD to improve their activity in cell culture.

### Saint PhD Increases Stapled Peptide Activity by Enhancing Endosomal Escape

**Figure 5:**
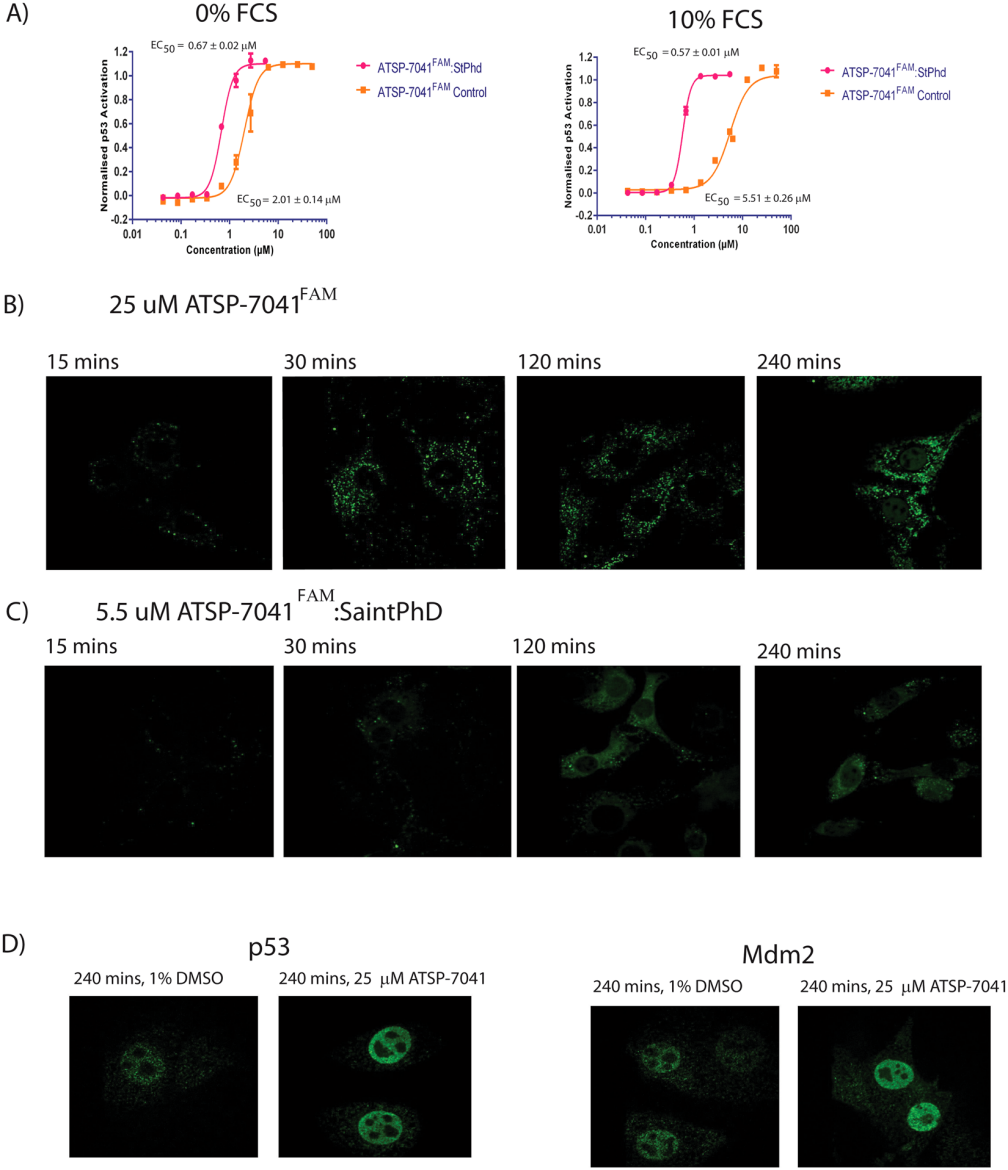
(**A**) ATSP-7041^FAM^ either formulated with Saint PhD or non-formulated was titrated onto T22 p53 reporter cells in the presence or absence of serum and p53 activity determined. (**B**) Time course experiment showing cellular uptake of non-formulated ATSP-7041^FAM^ at a peptide concentration of 25 µM into T22 cells in the presence of 10% FCS, which have been pulse labeled at different time intervals. (**C**) Time course experiment showing cellular uptake of ATSP-7041^FAM^:SAINTPhD at a peptide concentration of 6 µM into T22 cells with 10% FCS present, which have been pulse labeled at different time intervals. (**D**) Fixed T22 cells were stained for Mdm2 and p53 protein levels after 4 hour treatment with either 1% DMSO or 25 uM ATSP-7041 in the presence of 10% FCS.

To further understand the precise mechanism of improvement of VIP-82:SaintPhD over unformulated peptide we decided to incorporate a fluorescein (FAM) fluorophore into the design of the stapled peptide to allow examination of the cellular distribution of the peptide. Fluorescein labelled peptides were synthesized for VIP-82, ATSP-7041 and sMTIDE-02, by addition of FAM to their N-terminus via a β-alanine linker, and then tested in the T22 p53 reporter assay to ensure that they retained comparable biological activity to their unlabeled analogs, respectively (Figure S3). sMTIDE-02^FAM^ displayed no activity under high serum conditions and only marginal activity in the absence of serum, whilst the determined EC50s for VIP-82^FAM^ were attenuated 2.5 fold and 4 fold, respectively, with regards to VIP-82. However, ATSP-7041^FAM^ retained comparable activity in the T22 p53 reporter assay to its unlabeled analogue ATSP-7041. ATSP-7041^FAM^ was then reformulated with Saint PhD, where in contrast to ATSP-7041, significant improvements were observed in the EC50s of p53 activation over the unformulated peptide (Fig. 5A), further underlining how small molecular changes influence the biological properties of the resulting SaintPhd:peptide lipocomplex. ATSP-7041^FAM^ was therefore used to examine the effects of SaintPhD on the cellular distribution and uptake of stapled peptides.

Live T22 cells under high serum conditions were pulse labelled with either ATSP7041^FAM^:Saint PhD (6 µM) or unformulated peptide (25 µM) at various times points and examined using confocal microscopy (Fig. 5B and C). These concentrations were selected as they induced maximal activity of p53. ATSP7041^FAM^:Saint PhD and free ATSP7041^FAM^ were both initially internalized into the cell via cytoplasmic vesicles, where upon at later time points dramatic differences can be seen in the distribution of the peptide (Fig. 5B and C). In the case of ATSP-7041^FAM^ staining of the vesicles then becomes more intense over time followed by the accumulation of the FAM labelled peptide in the nucleus. In contrast, ATSP7041^FAM^:Saint PhD is rapidly released from the vesicles into the interior of the cell resulting in a diffuse cytoplasmic and nuclear stain. These results clearly demonstrate that the Saint PhD lipid formulation is enhancing vesicular escape of the stapled peptide and enabling higher intracellular levels of ATSP-7041^FAM^ to be reached at much lower treatment concentrations when compared to stapled peptide alone. In addition the staining of the nucleus by formulated and unformulated peptides highlights that ATSP-7041^FAM^ reaches the correct cellular locality to exert its functional activity. This is further supported by the detection of increased nuclear protein expression of both Mdm2 and p53 (Fig. 5D). The SaintPhD lipid formulation consists of 2 principal lipid components, the pyridinium cationic amphiphile Saint and the phospholipid DOPE (1,2-dioleoyl-*sn*-glycero-3-phosphoethanolamine), which is neutrally at physiological pH. DOPE is a well-known endosomolytic reagent^30^ and is most likely responsible for the increase in potency of the VIP-82:SaintPhD lipo-complexes with respect to unformulated VIP-82, after internalization of the complex into the cell. DOPE is a zwitterionic lipid, which under the acidic conditions of late endosomes undergoes a lipid phase change from lamellar to the inverse hexagonal phase that is disruptive to cellular lipid membrane bilayers^31–33^, enabling VIP-82 to undergo endosomal escape.

## Conclusion

The covalent modification of peptides using techniques such as stapling in order to stabilize their secondary structure represents an exciting opportunity to develop novel biologically active peptides that target intracellular interactions^9^. In this work we demonstrate that stapled peptides can be identified which interact with Mdm2, reactivate p53 and induce no LDH release under high or low serum conditions. We also confirmed the cell permeability of stapled peptides and their mechanism of p53 reactivation through the use of a live cell nanoBRET intracellular Mdm2:p53 interaction assay. In addition we ascertained that under conditions where stapled peptides induced LDH leakage that p53 re-activation and complex disruption occurred at much lower concentration thresholds, indicating that membrane destabilization is not a stress activator of the p53 pathway. The observation that p53 activity also does not occur with peptides where Mdm2 binding is abrogated but membrane disruption still takes places further corroborates this.

However, despite the isolation of a stapled peptide that caused minimal membrane perturbation with confirmed intracellular activity, only a marginal improvement in the potency of p53 activation was gained. This was overcome by screening VIP-82 with various lipid based formulations, ranging from micellular to liposomal, and identifying a system that prevented sequestration by serum and enhanced cellular uptake. Excitingly this enhancement in cellular uptake resulted in an order of magnitude improvement in biological activity as measured with p53 reporter cell line. Fluorescence studies further revealed that the main mechanism of this improvement by SaintPhD was vesicular escape (Fig. 5B and C), which enabled the stapled peptide to more efficiently enter the cytoplasm and disrupt the p53:Mdm2 complex. However, not all the stapled peptides formulated with this lipid based system showed similar levels of enhancement, indicating that the potential synergy of the formulation with the compound is sequence dependent. This raises a new avenue of development where peptides can be designed in conjunction with lipid based delivery systems to improve their biological properties rather than through iterative rounds of peptide design and staple optimization for marginal gain. In addition the earlier application of lipid systems to stapled peptide development will allow more rapid identification of conditions that prevent peptide aggregation and improve their solubility, lowering the barrier of entry into animal studies.

The assays outlined in this study provide a robust framework for the identification and validation of stapled peptides that disrupt specific intracellular complexes. The results presented here should re-invigorate research into stapled peptides and further enable their transition into the clinic. The application of new delivery technologies, such as lipid formulation, to improve the biological activity and biochemical properties of stapled peptides represents a new and exciting frontier in the development of these compounds.

## Materials and Methods

### Peptide Synthesis and Purification

See supplementary information.

### Mdm2 (1–125) protein Expression and Purification

See supplementary information.

### Mdm2 Competitive Fluorescence Anisotropy Assay and K_d_ Determination

See supplementary information.

### Preparation of Stapled Peptide Stock and Working Solutions

See Supplementary information.

### p53 Gene Reporter Assays

T22 cells, which were stably transfected with a p53 responsive β-galactosidase reporter, were seeded into a 96-well plate at a cell density of 8000 cells per well. Cells were also maintained in Dulbecco’s Minimal Eagle Medium (DMEM) with 10% fetal calf serum (FCS) and 1% (v/v) penicillin/streptomycin. The cells were incubated for 24 hours and followed by cell media removal and replacement with 90 µl of fresh DMEM either containing 10% FCS or 0% FCS. 10 µl of each compound was then added directly to each well and incubated for 18 hours. Final working concentration of DMSO was 1% v/v. Corresponding negative control wells with 1% DMSO only were also prepared. β-galactosidase activity was detected using the FluoReporter LacZ/Galactosidase Quantitation kit (Invitrogen) as per manufacturer’s instructions. Measurements were carried out using an Envision multiplate reader (Perkin-Elmer). Maximum p53 activity was defined as the amount of β-galactosidase activity induced by 25 µM Nutlin. This was was then used to normalize the experimental results.

### Lactate (LD) Dehydrogenase Release Assay

T22 cells or HEK 293 cells were seeded into a 96-well plate at a cell density of 5000 cells per well. Cells were maintained in Dulbecco’s Minimal Eagle Medium (DMEM) with 10% fetal bovine serum (FBS) and 1% (v/v) penicillin/streptomycin. The cells were incubated for 24 hours followed by cell media removal and addition of 90 µl of DMEM either with or without 10% FCS. Cells were then treated with compounds/peptide for 2 hours in DMEM with or without 10% FBS. Final working concentration of DMSO was 1% v/v. Corresponding negative control wells with 1% DMSO only were also prepared. Cytosolic lactate dehydrogenase release was detected using the cytoTox 96® non-radioactive cytotoxicity Assay kit (Promega) as per manufacturer’s instructions. Measurements were carried out using an Envision multiplate reader (Perkin-Elmer). Maximum LDH release was defined as the amount of LDH released when cells were lysed in the presence of 0.1% TRITON X-100 and was used to normalize the results.

### Peptide Formulation

See supplementary information.

## Electronic supplementary material

Supplementary Info
