# Supplementary material for: Enhancing Specific Disruption of Intracellular Protein Complexes by Hydrocarbon Stapled Peptides Using Lipid Based Delivery
Source: Sci Rep. 2017 May 11;7:1763. doi: 10.1038/s41598-017-01712-5 (PMC5431883; doi:10.1038/s41598-017-01712-5)
Supplement: Supplementary file 1 — Supplementary Info [file 41598_2017_1712_MOESM1_ESM.pdf]

Supplementary Information for:

# Enhancing Specific Disruption of Intracellular Protein Complexes by Hydrocarbon Stapled Peptides Using Lipid Based Delivery

**D Thean[1*], J Ebo[1,], T Luxton[1,*]Xue'Er Cheryl Lee[2], TY Yuen[2], FJ Ferrer[1], CW Johannes[2], DP Lane[1] and CJ Brown[1*]**

[1]p53 Laboratory, A*STAR, Singapore

[2] Organic Chemistry, ICES, A*STAR, Singapore

*****Corresponding Author (cjbrown@p53lab.a-star.edu.sg)

| Compound Name | Sequence | K$_d$ (nM) |
|---|---|---|
| (1) sMTIDE-02 | Ac-TSFR$_8$EYWALLS$_5$-NH$_2$ | 34.35 ± 2.0 |
| (2) VIP-62 | AC-TSF R$_8$EYWALLS$_5$K- TSFR$_8$EYWALLS$_5$-NH$_2$ | 528.5 ± 32.5 |
| (3) VIP-63 | Ac-TSFR$_5$EYWR$_5$LLSENF-NH$_2$ | 11.6 ± 0.9 |
| (4) VIP-64 | Ac-TSFR$_5$EYWR$_5$LLSENY-NH$_2$ | 8.8 ±0.6 |
| (5) VIP-65 | Ac-TSFR$_8$EYWALLS$_5$ENF-NH$_2$ | 19.7 ± 1.3 |
| (6) VIP-66 | Ac-TSFR$_8$EYWALLS$_5$ENY-NH$_2$ | 23.1 ± 1.5 |
| (7) VIP-67 | Biotin-Ahx-TSF R$_8$EYWALLS$_5$-NH$_2$ | 100.2 ± 7.7 |
| (8) VIP-68 | Biotin-Ahx-TSF R$_8$EYWAL(CBA)S$_5$-NH$_2$ | 41.6 ± 1.7 |
| (9)VIP-72 | Ac-TSFR$_8$EYWALLB$_5$-NH$_2$ | >10,000 |
| (10) VIP-73 | MYR-Ahx-Ahx-TSFR$_8$EYWALLS$_5$-NH$_2$ | >10, 000 |
| (11) VIP-71 | Ac-TSFR$_8$EYWAL(CBA)S$_5$E-NH$_2$ | 14.0 ± 1.1 |
| (12) VIP-76 | Ac-TSF(AIB)EYWALL(AIB)E-NH$_2$ | 35.9 ± 2.3 |
| (13) VIP-77 | Ac-KK-Ahx- TSFR$_8$EYWALLS$_5$-NH$_2$ | 46.8 ± 3.9 |
| (14) VIP-78 | Ac-RRR-Ahx- TSFR$_8$EYWALLS$_5$-NH$_2$ | 78.8 ± 8.2 |
| (15) VIP-79 | Ac-HHH-Ahx- TSFR$_8$EYWALLS$_5$-NH$_2$ | 130.1 ± 11.7 |
| (16) VIP-80 | Ac-RRR-Ahx-TSFR$_8$EYWALLS$_5$ENF-NH$_2$ | 45.0 ± 4.1 |
| (17) VIP-81 | Ac-HHH-Ahx-TSFR$_8$EYWALLS$_5$ENF-NH$_2$ | 61.2 ± 6.5 |
| (18) VIP-82 | Ac-KK-Ahx-TSFR$_8$EYWALLS$_5$ENF-NH$_2$ | 24.4 ± 2.3 |
| (19) VIP-83 | Biotin-Ahx- TSFR$_8$EYWALLS$_5$ENF-NH$_2$ | 24.6 ± 2.2 |
| (20) VIP-85 | Ac-RRR-Ahx-TSFR$_8$EYWALLS$_5$ENY-NH$_2$ | 44.9 ± 3.5 |
| (21) VIP-86 | Ac-KK-Ahx-TSFR$_8$EYWALLS$_5$ENY-NH$_2$ | 23.5 ± 1.4 |
| (22) VIP-87 | Biotin-Ahx- TSFR$_8$EYWALLS$_5$ENY-NH$_2$ | 46.2 ± 3.3 |
| (23) YS01 | Ac-TSFR$_5$EYWR$_5$LLPENF-NH$_2$ | 9.9 ± 1.5 |
| (24) YS02 | Ac-TSFR$_5$EYWR$_5$LLPENY-NH$_2$ | 7.4 ± 1.5 |
| (25) VIP-115 | Ac-KKK- Ahx-TSFR$_8$EYWALLS$_5$ENF-NH$_2$ | 11.6 ± 1.2 |
| (26) VIP-116 | Ac-K- Ahx-TSFR$_8$EYWALLS$_5$ENF-NH$_2$ | 15.1 ± 1.7 |
| (27) VIP-117 | Ac-RR- Ahx-TSFR$_8$EYWALLS$_5$ENF-NH$_2$ | 160.0 ± 14.6 |
| (28) VIP-118 | Ac-R- Ahx-TSFR$_8$EYWALLS$_5$ENF-NH$_2$ | 39.5 ± 3.9 |
| (29)VIP-119 | Ac-KK- Ahx-LTFR$_8$EYWALLS$_5$ENF-NH$_2$ | 2.7 ± 0.9 |
| (30) VIP-120 | Ac-LTFR$_8$EYWALLS$_5$ENF-NH$_2$ | 1.8 ± 0.7 |
| (31) VIP-121 | Ac-TSFR$_8$EYWALLS$_5$-NH$_2$ (azridinated) | 83.2 ± 9.7 |
| (32) VIP-122 | Ac-KLAK-Ahx-TSFR$_8$EYWALLS$_5$-NH$_2$ | 3.7 ± 0.9 |
| (33) VIP-124 | Ac-KK-Ahx-TSFR$_8$EYWAL(CBA)S$_5$ENF-NH$_2$ | 2.7 ± 0.4 |
| (34) VIP-125SCRAM | Ac-KK-Ahx-TSLR$_8$EYFALWS$_5$ENF-NH$_2$ | >10,000 |
| (35) VIP-126 SCRAM | Ac-TSLR$_8$EYFALWS$_5$-NH$_2$ | >10,000 |
| (36) VIP-127 SCRAM | Ac-KKK- Ahx-TSLR$_8$EYFALWS$_5$ENF-NH$_2$ | >10,000 |
| (37) VIP-128 SCRAM | Ac-RRR- Ahx-TSLR$_8$EYFALWS$_5$ENF-NH$_2$ | >10,000 |
| (38) VIP-129 | Ac-KLAK-Ahx-TSFR$_8$EYWALLS$_5$ENF-NH$_2$ | 9.1 ± 1.0 |
| (39) VIP-130 | Ac-K(K$_2$)-Ahx- TSFR$_8$EYWALLS$_5$-NH$_2$ | 33.5 ± 3.1 |
| (40) VIP-131 | Ac-K(Me$_2$)K(Me$_2$)-Ahx- TSFR$_8$EYWALLS$_5$ENF-NH$_2$ | 25.6 ± 2.6 |
| (41) VIP-132 | Ac-k-Ahx- TSFR$_8$EYWALLS$_5$ENF-NH$_2$ | 8.0 ± 0.8 |
| (42) VIP-133 | Ac-kk-Ahx- TSFR$_8$EYWALLS$_5$ENF-NH$_2$ | 10.0 ± 1.0 |
| (43) VIP-134 | Ac-kkk-Ahx- TSFR$_8$EYWALLS$_5$ENF-NH$_2$ | 6.2 ± 0.9 |
| (44) VIP-135 | Ac-KK-Ahx- TSFR$_8$EY($^{6\text{-Cl}}$W)ALLS$_5$ENF-NH$_2$ | 0.6 ± 0.3 |
| (45) VIP-136 | Ac-KK-Ahx- TSFR$_8$EY($^{6\text{-Cl}}$W)AL(CBA)S$_5$ENF-NH$_2$ | 0.6 ± 0.2 |
| (46)VIP-137 | Ac-TSFR$_8$EY($^{6\text{-Cl}}$W)ALLS$_5$E-NH$_2$ | < 1 * |
| (47)VIP-138 | Ac-TSFR$_8$EY($^{6\text{-Cl}}$W)AL(CBA)S$_5$E-NH$_2$ | <1 * |
| (48)VIP-139 | Ac-TSFR$_8$EY($^{6\text{-Cl}}$W)ALLS$_5$-NH$_2$ | 2.7 ± 0.5 |
| (49)VIP-140 | Ac-KK-Ahx-TSFR$_8$EY($^{6\text{-Cl}}$W)ALLS$_5$E- NH$_2$ | 0.4 ± 0.15 |
| (50)VIP-150 | Biot-SGSGTSFR$_8$EYWALLS$_5$- NH$_2$ | 54.2 ± 5.9 |
| (51)VIP-151 | Ac-KK SGSGTSFR$_8$EYWALLS$_5$- NH$_2$ | 33.6 ± 5.6 |
| (52) ATSP-7041 | Ac- LTFR$_8$EYWALQ(CBA)$_5$SAA-NH$_2$ | 7.5 ± 1.5 |

**Table S1:** Apparent dissociation constants determined using competitive fluorescence anisotropy experiments for compounds within the stapled peptide library. * denotes dissociation constants that cannot be accurately determined by the assay. R$_8$= (R)-2-(7'octenyl) alanine, S$_5$= (*S*)-2-(4'-pentenyl) alanine , CBA= cyclobutyl alanine , AIB = amino isobutyric acid , Ahx = aminohexanoic acid, K(K$_2$) = N$^6$,N$^6$-di-L-lysine-L-lysine, K(Me$_2$)= N$^6$,N$^6$-dimethyl-L-lysine.

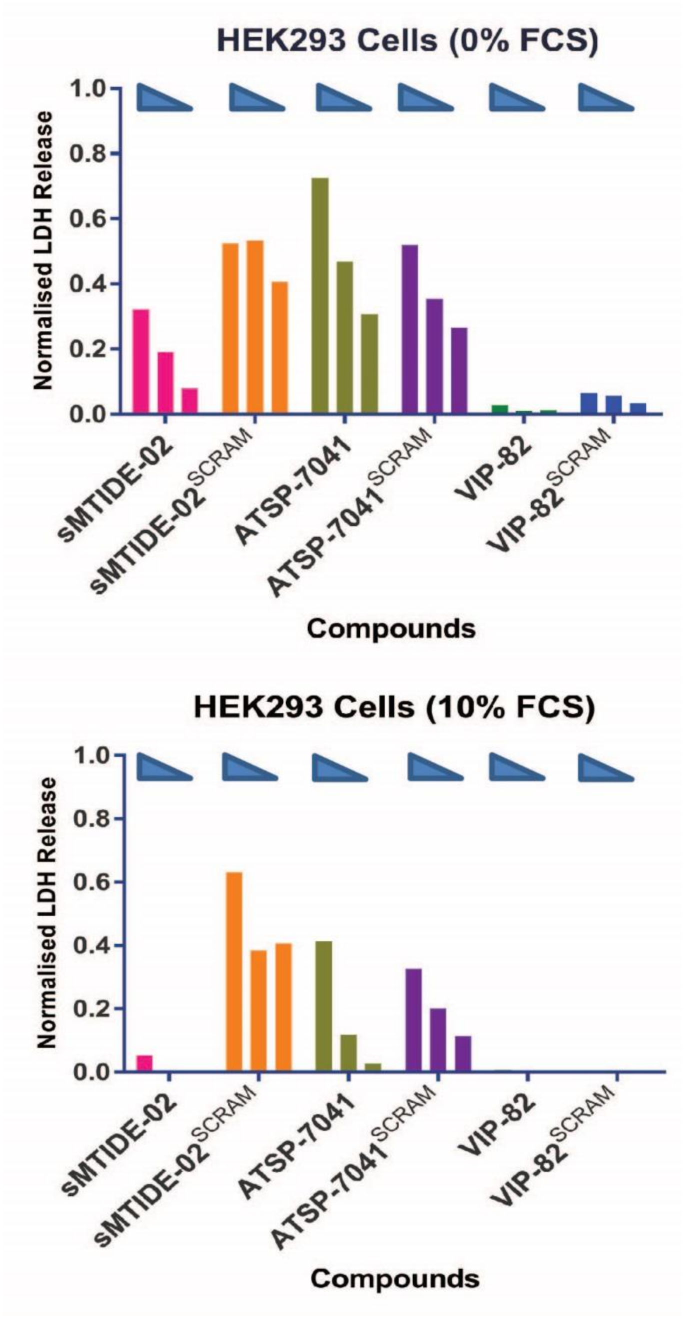


**Figure S1:** ATSP-7041, sMTIDE-02, VIP-82 and their scrambled analogues were screened for their ability to induce LDH release in HEK293 cells after a 2 hour treatment period in **A)** the absence of FCS and **B)** the presence of 10% FCS. Compounds were tested at concentrations of 12.5 µM, 25 µM and 50 µM. Results were normalized to maximal LDH release as measured by treatment with 0.1% Triton X100.

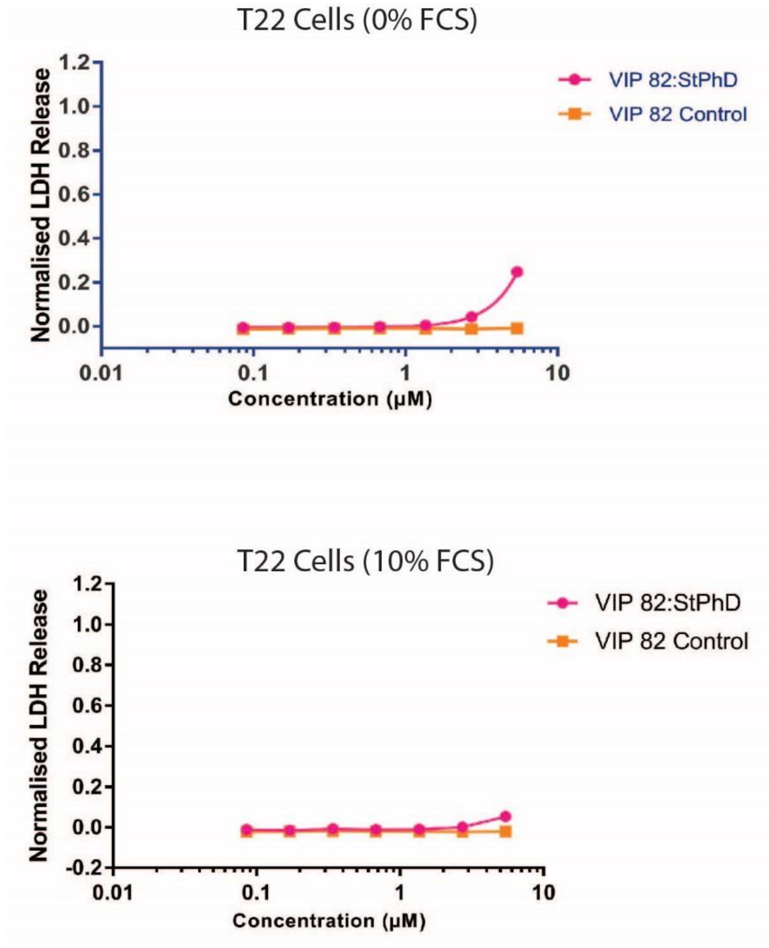


**Figure S2:** VIP-82 either formulated with Saint PhD or non-formulated was titrated onto T22 p53 reporter cells in the presence or absence of serum and LDH leakage levels assessed.

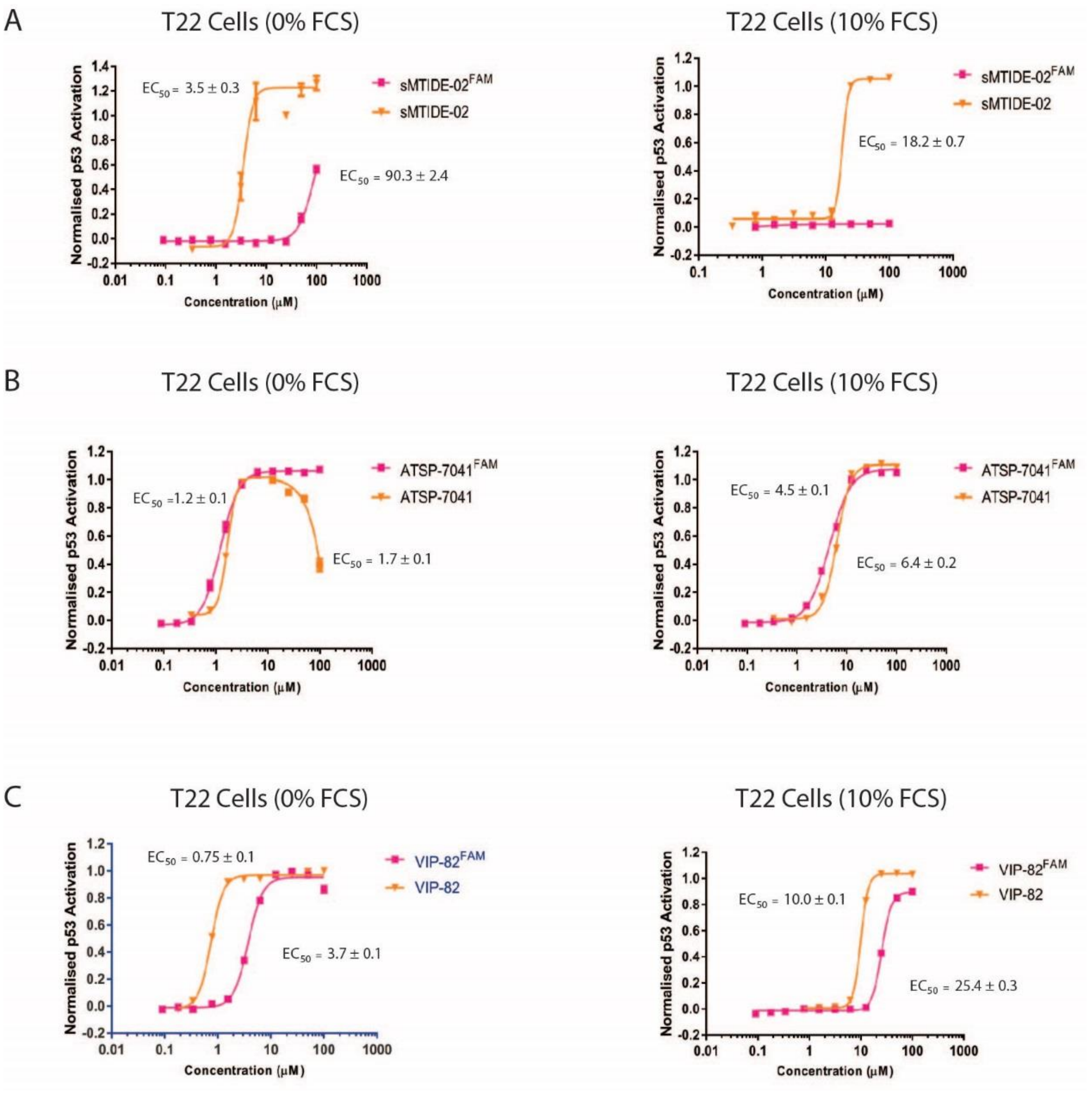


**Figure S3:** ATSP-7041, sMTIDE-02 and VIP-82 and their FAM labelled counterparts were titrated onto murine T22 p53 reporter cells in the presence and absence of serum and p53 activity assessed. EC50 values of p53 activation (µM) are denoted next to the corresponding titration curve.

**Peptide Synthesis**

Ramage Chemmatrix resin was obtained from PCAS-Biomatrix (Quebec, Canada). L-amino acids were obtained from Advanced Chemtech (Louisville, KY). Fmoc-threonine, serine, glutamic acid and tyrosine were t-butyl protected and Fmoc-tryptophan was not Boc protected. Unnatural alkenyl amino acids were purchased from OKeanos (Beijing, China). All other solvents and reagents were obtained from Sigma-Aldrich. 1,2-dichloroethane (DCE) was dried overnight over activated molecular sieves and purged with Argon for 30 min prior to use. All other reagents were used as received.

The peptides were synthesized by Fmoc chemistry on a Syro II peptide synthesizer (Biotage) at the 0.1 mmol scale using Ramage Chemmatrix resin (0.53 mmol/g). The dry resin was swelled with 1-methyl-2-pyrrolidinone (NMP) before use. The Fmoc protecting group was removed by treatment with 40% piperidine in NMP (3 min) followed by a second treatment with 20% piperidine in NMP (12 min). The Fmoc-protected amino acids (5 equiv.) were coupled using diisopropylcarbodiimide (DIC) as the activating agent (5 equiv.) and 1-aza benzotriazole (HOAt) as the additive in NMP (0.5 M). The coupling time was 90 min for all amino acids except for (*S*)-N-Fmoc-2-(4'-pentenyl)alanine and (*R*)-N-Fmoc-2-(7'-octenyl)alanine ($\mathbf{S_5}$ and $\mathbf{R_8}$ respectively). $\mathbf{S_5}$ and $\mathbf{R_8}$ (4 equiv.) were manually pre-activated for 7 min and coupled to the peptide resin for two hours. Following deprotection of the final Fmoc group, the peptides were acetylated using a mixture of acetic anhydride/diisopropylethylamine/dimethylformamide (2/2/1) for 60 min. After each coupling, deprotection and acetylation reaction, the resin was thoroughly washed with NMP.

Ring-closing metathesis of resin-bound, N-acetylated peptides was performed manually using a 5 mg/mL solution of Grubbs I catalyst (20 mol%) in dry DCE at room temperature under an atmosphere of inert argon (3 x 2 h treatments). After the reaction, the solution was drained, the

resin washed with DCE (3 x 1 min), DMSO (1 x 2 h) and methanol (3 x 1 min) then dried *in vacuo* overnight. Cleavage of the peptide from the resin was achieved using 8 mL of TFA cocktail consisting trifluoroacetic acid/triisopropylsilane/water (95/2.5/2.5) for 2 h followed by filtration and precipitation with diethyl ether. The precipitate was collected by centrifugation, dried and redissolved in a 3:2 mixture of acetonitrile and water.

The pure peptides (>90% purity) were obtained by purification using a preparative HPLC system (Agilent) on a Jupiter C12 reversed-phase preparative column (Phenomenex, 4 μm, Proteo 90 Å, 250 x 10 mm). The peptides were characterized by LC-MS. Mass spectra were obtained by electrospray in positive or negative ion mode.

**Mdm2 (1-125) protein Expression and Purification**

Mdm2 (1–125)) was ligated into the GST fusion expression vector pGEX-6P-1 (GE Lifesciences) via a BAMH1 and NDE1 double digest. BL21 DE3 competent bacteria were then transformed with the GST tagged (1–125) Mdm2 fusion construct. A single colony was picked and transformed cells were grown in LB medium at 37°C to an OD600 of ~0.6 and induction was carried out with 1 mM IPTG at room temperature. Cells were harvested by centrifugation and the cell pellets were resuspended in 50 mM Tris pH 8.0, 10% sucrose and then sonicated. The sonicated sample was centrifuged for 60 mins at 17,000 g at 4°C. The supernatant was applied to a 5 ml FF GST column (Amersham) pre-equilibrated in wash buffer (Phosphate Buffered Saline, 2.7 mM KCL and 137 mM NaCL, pH 7.4) with 1mM DTT. The column was then further washed by 6 volumes of wash buffer. Mdm2 was then purified from the column by cleavage with Precission (GE Lifesciences) protease. 10 units of precission protease, in one column volume of PBS with 1mM DTT buffer, were injected onto the column. The cleavage reaction was allowed to proceed overnight at 4°C. The cleaved protein was then eluted off the column with wash buffer. Protein fractions were

analyzed with SDS page gel and concentrated using a Centricon (3.5 kDa MWCO) concentrator, Millipore. Mdm2 protein samples were then dialyzed into a buffer solution containing 20mM Bis-Tris, pH 6.5, 0.05M NaCl with 1mM DTT and loaded onto a monoS column pre-equilibrated in buffer A (20mM Bis-Tris, pH 6.5, 1mM DTT). The column was then washed in 6 column volumes of buffer A and bound protein was eluted with a linear gradient of 1M NaCL over 25 column volumes. Protein fractions were analyzed with SDS page gel and concentrated using a Centricon (3.5 kDa MWCO) concentrator, Millipore. The cleaved Mdm2(1-125) construct was then purified to 90% purity. Protein concentration was determined using A280 using an extinction coefficients of 10430 M-1cm-for Mdm2 (1–125).

**Mdm2 Competitive Fluorescence Anisotropy Assay and $K_d$ Determination**

Purified MDM2 (1-125) protein was titrated against 50 nM carboxyfluorescein (FAM)-labeled 12/1 peptide[24] (FAM-RFMDYWEGL-$NH_2$). Dissociation constants for titration of MDM2 against FAM-labeled 12/1 peptide were determined by fitting the experimental data to a 1:1 binding model equation shown below:[25,26]

$$r = r_o + (r_b - r_o) \times \frac{(K_d + [L]_t + [P]_t) - \sqrt{K_d + [L]_t + [P]_t)^2 - 4[L]_t[P]_t}}{2[L]_t}$$

Equation 1.

[P] is the protein concentration (MDM2), [L] is the labeled peptide concentration, r is the anisotropy measured, r0 is the anisotropy of the free peptide, $r_b$ is the anisotropy of the MDM2–FAM-labeled peptide complex, $K_d$ is the dissociation constant, $[L]_t$ is the total FAM labeled

peptide concentration, and $[P]_t$ is the total MDM2 concentration. The determined apparent $K_d$ value of FAM-labeled 12/1 peptide (13.0 nM) was used to determine the apparent $K_d$ values of the respective competing ligands in subsequent competition assays.

Apparent $K_d$ values were determined for a variety of molecules via competitive fluorescence anisotropy experiments. Titrations were carried out with the concentration of MDM2 held constant at 250 nM and the labeled peptide at 50 nM. The competing molecules were then titrated against the complex of the FAM-labeled peptide and protein. Apparent $K_d$ values were determined by fitting the experimental data to the equations shown below:[26,27]

$$r = r_o + (r_b + r_o) \times \frac{2\sqrt{(d^2 - 3e)}\cos(\theta/3) - 9}{3K_{d1} + 2\sqrt{(d^2 - 3e)}\cos(\theta/3) - d}$$

$$d = K_{d1} + K_{d2} + [L]_{st} + [L]_t - [P]_t$$

$$e = ([L]_t - [P]_t)K_{d1} + ([L]_{st} - [P]_t)K_{d2} + K_{d1}K_{d2}$$

$$f = -K_{d1}K_{d2}[P]_t$$

$$\theta = ar\cos\left[\frac{-2d^3 + 9de - 27f}{2\sqrt{(d^2 - 3e)^3}}\right]$$

Equation 2.

$[L]_{st}$ and $[L]_t$ denote labeled ligand and total unlabeled ligand input concentrations, respectively. $K_{d2}$ is the dissociation constant of the interaction between the unlabeled ligand and the protein. In

all competitive types of experiments, it is assumed that $[P]_t > [L]_{st}$, otherwise considerable amounts of free labeled ligand would always be present and would interfere with measurements. $K_{d1}$ is the apparent $K_d$ for the labeled peptide used in the respective experiment, which has been experimentally determined as described in the previous paragraph. The FAM-labeled peptide was dissolved in dimethyl sulfoxide (DMSO) at 1 mM and diluted into experimental buffer. Readings were carried out with an Envision Multilabel Reader (PerkinElmer). Experiments were carried out in PBS (2.7 mM KCl, 137mM NaCl, 10 mM $Na_2HPO_4$ and 2 mM $KH_2PO_4$ (pH 7.4)) and 0.1% Tween 20 buffer. All titrations were carried out in triplicate. Curve-fitting was carried out using Prism 4.0 (GraphPad).

To validate the fitting of a 1:1 binding model we carefully determined that the anisotropy value at the beginning of the direct titrations between MDM2 and the FAM-labeled peptide did not differ significantly from the anisotropy value observed for the free fluorescently labeled peptide. Negative control titrations of the ligands under investigation were also carried out with the fluorescently labeled peptide (in the absence of MDM2) to ensure no interactions were occurring between the ligands and FAM-labeled peptide. In addition we ensured that the final baseline in the competitive titrations did not fall below the anisotropy value for the free FAM-labeled peptide, which would otherwise indicate an unintended interaction between the ligand and the FAM-labeled peptide to be displaced from the MDM2 binding site.

**Nanobret Mdm2:p53 Cell Based Assay**

HEK293 cells were seeded at a cell density of 250,000 cells per well into a 6 well plate and incubated overnight at 37 ˚C and 5% $CO_2$ in DMEM with 0.3 mg/ml glutamine, 100 IU/ml penicillin, 100 μg/ml streptomycin and 10% fetal calf serum. Each well was transfected with a

1µg:1µg DNA mixture of NanoLuc-MDM2 fusion vector and p53-HaloTag fusion vector using Fugene HD following the manufacturer's instructions (PROMEGA). After a 20 hour overnight incubation period cell media was removed and the cells were washed with PBS saline. Cells were then trypsinised and re-suspended in Opti-MEM media with 10% FCS. Cells were then spun down at 1000 rpm for 5 minutes at room temperature. Supernatant was then discarded and cells re-suspended to a density of to $2.2x10^5$ cells per ml in Opti-MEM I reduced serum containing 10% FCS with no added red phenol. Cells were divided into 2 pools where either HaloTag NanoBRET 618 ligand was added to achieve a final concentration of 100 nM or DMSO was added to achieve a final concentration of 0.1% DMSO. 90 µl of cells labelled with the NanoBRET 618 ligand were added to the wells of a white opaque 96-well plate to be used for compound analysis. Either 10 µl of a 10% DMSO control in FPLC grade water or a suitable 2-fold dilution series of the compound under study in a 10-fold higher stock concentration (containing 10% DMSO and FPLC grade water solution) was added. Controls wells were also setup with 90 µl of non NanoBRET 618 ligand labelled cells with the addition of 10 µl of 10% DMSO in FPLC grade water. 96 well plates were then incubated for 4-6 hours at 37˚C, 5% $CO_{2.}$ 25 µl of a 100-fold dilution of Nano-Glo substrate (PROMEGA) in Opti-MEM containing no red phenol was added to each well and the plate shaken for 30 seconds. The donor emission (nanoLUC, 460 nm) and acceptor emission (NanoBRET 618 ligand, 619 nm) was measured within 10 minutes are addition of the Nano-Glo substrate using an Envision plate reader (PerkinElmer) within 10 minutes. Compound titrations in the absence of serum were performed using identical conditions with the exception that FCS was removed in the steps proceeding from the re-suspension of the freshly transfected HEK293 cells after trypsinisation. Nanobret values were derived from the emission and donor values using the following equation:

1) $618_{nm}$(Em)/$460_{nm}$(EM) = Raw NanoBRET Ratio = BU (BRET unit)

The raw BRET units were then converted to milliBRET units by multiplying by a thousand. Each concentration point in the compound titrations performed including controls was performed in triplicate. To correct for the donor contributed background the no acceptor mean (cells not labelled with HaloTAG 618) were subtracted from the experimental mean BRET values to derive the corrected nanoBRET ratio.

**Preparation of Stapled Peptide Stock and Working Solutions**

10 mM stock solutions of stapled peptides were prepared in 100% DMSO. Stapled peptides were then serially diluted in 100% DMSO and further diluted 10-fold into HPLC grade sterile water to prepare 10X working solutions in 10% DMSO/water of each compound at concentrations ranging from 1000 µM to 1.5 µM. Depending on the required volume used in the relevant assay, compounds were added to yield final concentrations ranging from 100 µM to 0.15 µM with a residual DMSO concentration of 1% v/v.

**Peptide Formulation**

**SaintPhD (Synvolux, Netherlands)**

Working compound solutions were prepared ranging (as described in **Preparation of Stapled Peptide Stock and Working Solutions)** from 100 µM to 1.5 µM in HEPES buffered saline (HBS) with a final DMSO concentration of 1% v/v. The resulting peptide solutions were then constituted in a 3:2 ratio with the SaintPhD reagent (provided by manufacturer) to yield a 10x working solution, and vortexed before use. This was then followed with incubation at room temperature for 5 minutes. Depending on the required volume used in the relevant assay, compounds were added to yield final peptide concentrations ranging from 6 µM to 0.05 µM with a residual DMSO

concentration of 0.06% v/v. Negative control treatments were performed by omitting addition of the SaintPhD mixture and replacing with HBS.  A negative control SaintPhD treatment was also performed with substitution of peptide component with HBS.

**Sterically Stabilised Micelles**

50 mg/mL of DSPE-mPEG2K (18:0) (purchased from Avanti Polar Lipids, Inc., USA) was dissolved in phosphate buffered saline (PBS) to form a stock solution of sterically stabilized micelles (SSM). Working peptide solutions were prepared by serially diluting the 10 mM stapled peptide stock solution (100% DMSO v/v) in DMSO to prepare peptide concentrations from 5 mM to 1.5 mM, in a addition to the starting 10 mM concentration. 10x working solutions of stapled peptide were prepared in a 1:1 ratio with the sterically stabilized micelles (SSMs) in PBS/10% DMSO v/v.  Depending on the required volume used in the relevant assay, compounds were added to yield final peptide concentrations ranging from 100 µM to 0.7 µM with a residual DMSO concentration of 1% v/v. Negative control treatments were performed by omitting addition of the SSM mixture and replacing with PBS.  A negative control treatment consisting of SSMs only was also performed with substitution of the peptide component with PBS.

**NanoCargo (Tecrea Ltd, UK)**

Working peptide solutions were prepared by serially diluting a 10 mM stapled peptide stock solution (100% DMSO v/v) in DMSO to prepare peptide concentrations from 5 mM to 1.5 nM, in addition to the starting 10mM concentration. Stapled peptides were then formulated with NanoCargo by dilution of 1 µl of peptide working solution (100% DMSO v/v) with 18 µl of phosphate buffered saline (PBS), followed by mixing with 1 µl of NanoCargo solution (provided by manufacturer).  The peptide:NanoCargo mixture was then further diluted by addition of 380 µl

of cell media (with or without 10% FCS). For addition to 96 well cell based assays, cell media was aspirated, followed by addition of 100 µl of peptide:Nanocargo mixture. Negative control treatments were prepared by omitting addition of the NanoCargo mixture and replacing with PBS. A negative control NanoCargo treatment was also constituted by replace of the peptide component with PBS.

**Live Cell Confocal Microscopy**

20,000 T22 p53 reporter cells were plated in 3.5 cm glass bottomed dishes in DMEM containing 10% FCS. After 24 h, the media was replaced with DMEM containing 10% FBS and treated with either 25 µM of ATSP-7041$^{FAM}$ or 6 µM of the ATSP-7041$^{FAM}$:SaintPhD lipocomplex at the following timepoints: 15 mins, 30 mins, 120 mins and 240 mins. T22 cells were then washed twice with PBD saline and media replaced with DMEM containing 10% FCS. Confocal images were acquired at the indicated time points using a Yokogawa CSU-22 spinning disk confocal built around a motorized Nikon Eclipse Ti microscope equipped with a stage-top incubator and $CO_2$ control system, equipped with a 100 × 1.4 NA Plan Apo objective lens, photometrics CoolSNAPHQ$^2$ camera and 491 nm laser. Acquisition parameters, shutters, filter positions and focus were controlled by MetaMorph software (Molecular Devices)).

**ImmunoFluorescence**

20,000 T22 p53 reporter cells were plated in 3.5 cm glass bottomed dishes in DMEM containing 10% FCS. After 24 h, the media was replaced with DMEM containing 10% FBS and treated with either 25 µM of ATSP-7041 or vehicle control for 4 hours. Cells were then washed with PBS and fixed with 4% formaldehyde in PBS, permeabilized with 4% formaldehyde/0.1% Triton X-100 in

PBS, and blocked with 50% FBS-6% milk-3% BSA-0.1% Triton X-100–0.05% $NaN_3$ in PBS. Independent cell samples were then incubated with either anti-p53 (CM5) (1:200) or anti-Mdm2 (2A10) (1:200), for 18 h at 4°C. Cells were washed with PBS and interrogated with secondary goat anti-rabbit/mouse IgG conjugated to Alexa Fluor 488 with regards to CM5 and 2A10, respectively, at room temperature. Samples were then washed in PBS and imaged using the same microscopy set up as described in the live cell confocal microscopy section.
